# Nrf2/HO-1 Signaling Stimulation through Acetyl-11-Keto-Beta-Boswellic Acid (AKBA) Provides Neuroprotection in Ethidium Bromide-Induced Experimental Model of Multiple Sclerosis

**DOI:** 10.3390/genes13081324

**Published:** 2022-07-25

**Authors:** Shubham Upadhayay, Sidharth Mehan, Aradhana Prajapati, Pranshul Sethi, Manisha Suri, Ayat Zawawi, Majed N. Almashjary, Shams Tabrez

**Affiliations:** 1 Division of Neuroscience, Department of Pharmacology, ISF College of Pharmacy, Moga 142001, Punjab, India; upadhayay.shubham11@gmail.com (S.U.); aradhanapjt@gmail.com (A.P.); pranshulsethiptl@gmail.com (P.S.); manishasuri9015@gmail.com (M.S.); 2 Department of Medical Laboratory Sciences, Faculty of Applied Medical Sciences, King Abdulaziz University, Jeddah 21589, Saudi Arabia; atzawawi@kau.edu.sa (A.Z.); malmashjary@kau.edu.sa (M.N.A.); 3 Vaccines and Immunotherapy Unit, King Fahd Medical Research Center, King Abdulaziz University, Jeddah 21589, Saudi Arabia; 4 Hematology Research Unit, King Fahd Medical Research Center, King Abdulaziz University, Jeddah 21589, Saudi Arabia; 5 Animal House Unit, King Fahd Medical Research Center, King Abdulaziz University, Jeddah 21589, Saudi Arabia; 6 King Fahd Medical Research Center, King Abdulaziz University, Jeddah 21589, Saudi Arabia

**Keywords:** multiple sclerosis, Nrf2/HO-1, acetyl-11-keto-beta-boswellic acid (AKBA), demyelination, ethidium bromide (Etb), neurodegeneration

## Abstract

Multiple sclerosis (MS) is a severe immune-mediated neurological disease characterized by neuroinflammation, demyelination, and axonal degeneration in the central nervous system (CNS). This is frequently linked to motor abnormalities and cognitive impairments. The pathophysiological hallmarks of MS include inflammatory demyelination, axonal injury, white matter degeneration, and the development of CNS lesions that result in severe neuronal degeneration. Several studies suggested downregulation of nuclear factor erythroid-2-related factor-2 (Nrf2)/Heme oxygenase-1 (HO-1) signaling is a causative factor for MS pathogenesis. Acetyl-11-keto-β-boswellic acid (AKBA) is an active *pentacyclictriterpenoid* obtained from *Boswellia serrata*, possessing antioxidant and anti-inflammatory properties. The present study explores the protective potential of AKBA on behavioral, molecular, neurochemical, and gross pathological abnormalitiesandhistopathological alterations by H&E and LFB staining techniques in an experimental model of multiple sclerosis, emphasizing the increase inNrf2/HO-1 levels in the brain. Moreover, we also examine the effect of AKBA on the intensity of myelin basic protein (MBP) in CSF and rat brain homogenate. Specific apoptotic markers (Bcl-2, Bax, andcaspase-3) were also estimated in rat brain homogenate. Neuro behavioralabnormalities in rats were examined using an actophotometer, rotarod test, beam crossing task (BCT),and Morris water maze (MWM). AKBA 50 mg/kg and 100 mg/kg were given orally from day 8 to 35 to alleviate MS symptoms in the EB-injected rats. Furthermore, cellular, molecular, neurotransmitter, neuroinflammatory cytokine, and oxidative stress markers in rat whole brain homogenate, blood plasma, and cerebral spinal fluid were investigated. This study shows that AKBA upregulates the level of antioxidant proteins such as Nrf2 and HO-1 in the rat brain. AKBA restores altered neurochemical levels, potentially preventing gross pathological abnormalities during MS progression.

## 1. Introduction

Multiple sclerosis (MS) is an immune-mediated neurological disease that causes acute inflammation, oligodendrocyte destruction, demyelination, andaxonal damage in the central nervous system, resulting in sclerotic lesions [1,2]. The clinical symptoms of MS progression are sensory, visual, and motor function impairments and autonomic dysfunction [3,4]. According to the most recent edition of the *Epidemiological Atlas of MS*, 2.8 million people worldwide have MS. Based on the current MS International Federation, India has around 145 thousand MS sufferers, with a prevalence rate of 11 per 100 thousand (https://www.atlasofms.org/map/india/epidemiology/number-of-people-with-ms (accessed on 19 June 2020)), with women being more vulnerable than men [5].

The pathophysiological process of MS is triggered by inflammatory cytokines such as CD4^+^T-helper cells, CD8^+^T-cells, CD20^+^B-cells, macrophages, monocytes, natural killer cells, and other pro-inflammatory cytokines [6,7]. CD8andT cells in MS patients’ white matter lesions are persistently activated; T cells destroy the CNS myelin layer and increase axonal demyelination, resulting in motor function loss [8]. Several pre-clinical animal models have investigated MS pathogenesis [9,10]. 

Demyelination with toxic substances helps to study the cellular and molecular changes during MS progression [11]. Ethidium bromide (EB) is a well-known toxin that induces demyelination via intracerebropeduncle injection, leading to molecular, behavioural, and neurochemical alterations in experimental rats [1,12]. EB spreads throughout the brain, causing oligodendrocyte destruction and axon demyelination, affecting motor and sensory functions [13]. Stereotaxic injection of EB into the cerebellar peduncle area of white matter tracts in the CNS causes microglia activation, oligodendrocyte destruction, and demyelination of most axons [1,14]. Several autoimmune disorders associated with chronic inflammation lead to excessive reactive oxygen species (ROS) generation in the CNS [15]. An accumulative study indicated that oxidative stress plays an important factor in MS etiology. In both MS and its experimental paradigms, excessive ROS production leads to oxidative stress, which has been identified as a major regulator of oligodendrocyte destruction and axonal demyelination [16].

In humans, Nrf2 is a transcription factor gene often present in the cytoplasm after binding to the Kelch-like ECH-associated protein-1 (Keap1) [17]. When exposed to ROS, Nrf2 is disrupted and transferred into the nucleus, where it attaches to the antioxidant response element (ARE) and stimulates the antioxidant enzyme hemeoxygenase-1 (HO-1) [18]. HO-1 plays an important role in maintaining immune homeostasis, and its disruption leads to immune-mediated inflammatory disorders such as MS [19]. Dysregulation of the Nrf2/HO-1 signaling in the CNS leads to oxidative stress-mediated neuroinflammation involved in the progression of autoimmune diseases like MS [20,21,22]. 

According to previous pre-clinical evidence, prolonged Nrf2/HO-1 downregulation initiates the development of several neurological disorders, including amyotrophic lateral sclerosis (ALS) [23], Alzheimer’s disease (AD) [24], Parkinson’s disease (PD) [25], and Huntington’s disease (HD) [26]. In addition, it is also involved in the pathogenesis of cerebral ischemia injury and intracerebral hemorrhage (ICH) [27,28]. Nrf2 is a transcription factor that controls the progression of various human diseases, including cancer [29]. Nrf2 activation may prevent cancer development and progression in healthy cells [30]. Nrf2 activators, such as vitamin E, protect against cadmium-induced hepatic toxicity [31], while sulforaphane prevents angiotensin II-induced cardiomyopathy [32] and autosomal dominant polycystic kidney disease [33]. Sulforaphane and cinnamaldehyde as Nrf2 activators enhance wound healing against streptozotocin-induced diabetes [34]. Previous studies suggest that Nrf2/HO-1 activators show neuroprotective action in MS patients when treated with dimethyl fumarate [35]. The Nrf2/HO-1 activator is an effective therapy for amyotrophic lateral sclerosis [36], Alzheimer’s disease [37], Parkinson’s disease [38], Huntington’s disease [39], and intracerebral hemorrhage [40].

Acetyl-11-keto-β-boswellic acid (AKBA) is a pentacyclic triterpenoid obtained from *Boswellia serrata,* which belongs to the frankincense family, with a yellowish-brown oleo-gum resin [41,42]. AKBA was found to possess antioxidant and anti-inflammatory properties [43] and is used for treating glioblastoma tumour cells, skin keratinocytes, myocardial injury, and osteoclastogenesis [44,45,46,47]. 

AKBA has shown neuroprotective effects on various diseases such as AD [48], PD [49], and ischemia-reperfusion injury [50]. AKBA has been shown to improve memory and cognitive dysfunction [51]. It also has a protective effect on sciatic nerve injury [52]. AKBA inhibits the differentiation of IL-17-producing CD4 + T (Th17) cells and plays a role in the etiology of multiple sclerosis [53,54]. AKBA, as an Nrf2/HO-1 activator, has a protective effect on cerebral ischemia [50,55].

Thus, the present study investigates the role of Nrf2/HO-1 downregulation in the development of experimental MS. We investigated the protective role of AKBA on behavioural, neurochemical, and morphological characteristics in ethidium bromide-treated MS-like rats. AKBA shows a protective effect in MS rats by upregulation of the Nrf2/HO-1 signaling pathway, which was confirmed by studying neurochemical parameters in biological samples such as CSF, blood plasma, and brain homogenates. Our research indicates that AKBA, as an Nrf2/HO-1 activator, could be a potential therapy option for people with MS and other neurodegenerative illnesses.

## 2. Materials and Methods 

### 2.1. Experimental Animals

During this research, a total of 36 rats were utilized. All trials were carried out on adult Wistar rats six months old and weighed between 250 and 300 g. These rats were obtained from the Central Animal House at the ISF College of Pharmacy in Moga, Punjab, India. The animals were housed in a chamber between 22–25 degrees and had a light-dark cycle. They also had unrestricted access to food and water. The Institute for Animal Ethics Committee (IAEC) funded and approved the project as 816/PO/ReBiBt/S/04/CPCSEA as IAEC/CPCSEA/Meeting No: 27/2020/Protocol No. 457, in compliance with the guidelines established by the government of India. The authors express their gratitude and acknowledgement towards Chairman, Mr. Parveen Garg, and Director-cum-Principal, Dr. G.D. Gupta, ISF College of Pharmacy (An Autonomous College), Moga (Punjab), India, for their incredible vision and support. Before the experiments, the animals had time to adapt to the laboratory environment.

### 2.2. Drugs and Chemicals

EB was obtained from Sigma-Aldrich (St. Louis, MO, USA). BAPEX, India, contributed an exgratia sample of AKBA. In addition, only analytical-grade compounds were employed in the study. Before use, the medications and chemicals were prepared in a clean, sterile environment. AKBA was dissolved in physiological saline and taken orally [36,48].

### 2.3. Protocol Schedule of Animal Experimentation

**Figure 1:**
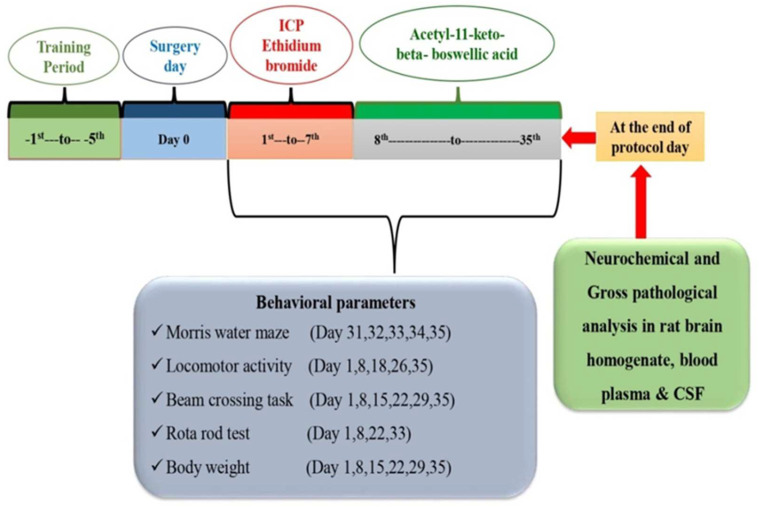
Experimental protocol schedule.

The investigation lasted 35 days in all. The rat brain’s intracerebropeduncle (ICP) area was injected with ethidium bromide (EB) from day 1 to day 7. From day 8 to the end of the experiment, AKBA was given continuously (35 days). Six groups of animals (*n* = 6) were randomly assigned. Group 1 was a vehicle control, Group 2 was a sham control, Group 3 was an AKBA perse (100 mg/kg, p.o.), Group 4 was assigned to EB (10 μL, ICP), Group 5 was assigned to EB (10 μL, ICP) + AKBA (50 mg/kg, p.o.), and Group 6 was assigned to EB (10 μL, ICP) + AKBA (100 mg/kg, p.o). The researchers in this study were unblinded, and their expertise in animal welfare was well-known. Various behavioral tests were performed from the first to the 35th day. Biological samples (blood plasma and cerebrospinal fluid) from adult Wistar rats were taken on day 36. Later, sodium pentobarbital (270 mg/mL, i.p.) was used to deeply anaesthetize the animals so they could be dissected for fresh brain tissue for biochemical analysis. As ummary of the study’s protocol may be found in Figure 1.

### 2.4. Experimental Animal Model of ICP-EB-Induced Experimental MS Rats

The EB-induced MS rat model was developed using the validated approach by Kumar et al. [1] and Sharma et al. [15]. For seven days in a row, rats were given an EB-ICP injection of 10 µL of 0.1 percent. The findings by Kumar et al. [1] revealed that EB induces neurological damage similar to an experimental animal model of MS and is regarded as a suitable paradigm for examining pathophysiological alterations similar to those of MS.

Rats were accustomed to the environment in the laboratory. Rats were anaesthetized intraperitoneally with 75 mg/kg ketamine after acclimatization. Rats were then placed in a flat position with their skulls in the stereotaxic apparatus. Before beginning the surgical operation, the bregma and lambda coordinates were aligned at the same height. The scalps of the rats were shaved, washed with 70% ethanol, and incised mid-sagittally with a scalpel. The skin was then pulled back, exposing the exposed skull where bregma and lambda were noted to assist in finding the coordinates for ICP injection. Rats’ eyes were wiped with cotton swabs dipped in normal saline, and bleeding was stopped with cotton buds. After drilling a hole with stereotaxic coordinates of AP = −11.4, ML = +2.6, and DV = −7.07, we put a cannula into the burr hole and sealed it with an ear-pin made of plastic. Following the placement of dental cement in the gaping wound, an absorbable surgical suture attached to a sterile surgical needle was used to close the wound. EB in sterile 0.9 percent saline was used to unilaterally produce demyelination by injecting ten microliters directly into the brain [1,15,56].

To provide post-operative care for the rats, each animal was given a polyacrylic cage, which was often covered in a warm cloth. They were given extra attention until they could move on their own two to three hours after being under anaesthesia. The temperature in the room was kept at a constant 25.3 °C. After surgery, the animals were kept in their cages for 2–3 days with milk and glucose water to minimize physical harm. Lignocaine gel was injected into the sutured area to alleviate discomfort. Gentamycin was administered intraperitoneally for three days to prevent sepsis. As a precaution, Neosporin powder was sprayed on their skin. Dehydration and other clinical signs such as fatigue were closely examined following surgery.

### 2.5. Parameters

#### 2.5.1. Measurement of Weight Variations

Throughout the investigation, the researchers took weight measurements every day at 1, 8, 15, 22, 29, and 35 days [57].

#### 2.5.2. Measurement of Relative Brain-Body Weight Ratio

The 35-day study methodology calculated the relative brain-body weight ratio [58].

### 2.6. Assessment of Behaviour Parameters

#### 2.6.1. Morris Water Maze Task

The Morris water maze (MWM) test assessed memory and cognitive impairment. During the experiment protocol schedule’s 31st, 32nd, 33rd, and 34th days, escape latency (ELT) was recorded using the MWM. Escape latency was defined as the time (seconds) it took rats to reach the target platform. On day 35, rats were placed in a swimming tank, and the time spent in the target quadrant (TSTQ) was counted for 120 s. The TSTQ measures the memory consolidation that occurred following the learning experience [59].

#### 2.6.2. Locomotor Activity

Locomotive activities were evaluated on an experimental schedule of 1, 8, 18, 26, and 35 days. Five minutes of locomotive activity were measured with an actophotometer to assess motor coordination, and results were represented as counts per five minutes [60].

#### 2.6.3. Beam Crossing Task

An evaluation of gait disturbances and foot slips was carried out using the beam crossing task test. At 1, 8, 15, 22, 29, and 35 days, the ability of each animal’s motor coordination was assessed. Over two minutes, the number of foot slips that occurred in each experiment was counted [61].

#### 2.6.4. Rotarod Test

Rotarod testing was used to examine motor coordination. The rotarod activity was carried out on days 1, 8, 22, and 33 of the protocol. The speed remained steady to a maximum of 15 rpm. Each rat’s fall-off time (seconds) was recorded for five minutes [62].

### 2.7. Neurochemical Parameters

#### 2.7.1. Collection and Preparation of Biological Samples

The rats were deeply anaesthetized with chloroform on day 36 before samples were collected. After anaesthesia, a fresh capillary tube was inserted under the nictitating membrane at the medial canthus of the eye, and the sinus was punctured. This retrobulbar puncture was used to obtain 1 to 2 millilitres of blood from the rats [63]. Centrifugation at 10,000× *g* for 15 min separated the plasma from the blood samples. The plasma was carefully preserved in a deep freeze (−80 °C) for subsequent neurochemical investigation.

Sodium pentobarbital (270 mg/mL, i.p.) was used to anaesthetize the rats following blood collection. A skin incision was done, and a translucent duramater was uncovered when the rats’ heads were held in a holder to reveal the arachnoid membrane. Inserting a 30-gauge needle into the cisterna magna at a 30° angle yielded a maximum amount of 100 µL CSF. The material was centrifuged at 2000× *g* for 10 min at 4 °C within 20 min of collection. When the supernatant was centrifuged, it was stored at −80 °C for subsequent examination [64,65].

As part of the study’s design, rats were decapitated to collect their CSF, and then their brains were removed and homogenized in the presence of 0.1 M (*w*/*v*)of chilled phosphate-buffered saline (pH = 7.4). It was centrifuged for 15 min at 10,000× *g*, the supernatant was separated, and the aliquots were frozen at −80 °C for subsequent biochemical analyses.

#### 2.7.2. Assessment of Cellular and Molecular Markers

To measure the amounts of Nrf2 in rat brain homogenate [36]and CSF [66], an ELISA kit was used. The levels of HO-1 in rat brain homogenate [36] and CSF sample [67] were determined using ELISA assay kits from Elabscience Biotechnology Inc. in China (Elabsciences, Wuhan, China), according to the manufacturer’s instructions. ELISA kits analysedMBP levels in rat brain homogenate [1] and cerebral spinal fluid [64].

#### 2.7.3. Assessment of Apoptotic Markers

ELISA kits were used to assess caspase-3 concentrations in brain homogenate [68,69]. A brain homogenate sample were tested for Bax protein levels [70,71]. A commercial ELISA kit was used to quantify the anti-apoptotic protein Bcl-2 levels in brain homogenate [71,72] (Elabs-sciences, Wuhan, China).

#### 2.7.4. Assessment of Neurotransmitter Levels

The striatal tissue sample was tested for dopamine levels. Ng/mg protein was used to measure the concentration of dopamine in rat brain homogenates [59]. In tissue samples, glutamate was measured using Alam and coworkers’ method after derivatization with o-phthalaldehyde/-mercaptoethanol (OPA/-ME). The ng/mg protein represents the glutamate concentration in rat brain homogenate [62,73]. A diagnostic kit was used to assess acetylcholine levels (Krishgen diagnostics, India). The kit’s instructions were strictly followed when preparing the samples and reagents. At 540 nm, the optical density of the reaction mixture was determined. The supernatant contained ng/mg protein of the neurotransmitter [74,75]. With the help of an electrochemical detector and a C18 reverse-phase column, researchers employed HPLC to quantify serotonin levels in the blood. In the mobile phase, acetonitrile (87:13, *v*/*v*) was mixed with sodium citrate buffer (pH 4.5). Before being injected into the sample injector, the supernatant was filtered via 0.22 mm nylon filters. A standard curve was constructed using a standard solution containing 10–100 mg/mLof serotonin [72].

#### 2.7.5. Measurement of TNF-α and IL-1β Levels

An immunoassay kit for rat brain homogenate [76,77]and blood plasma was used to measure TNF-α levels [78,79] (KRISHGEN BioSystem, Mumbai, India). Rat brain homogenate and blood plasma were used to measure the activity of IL-1β [80,81].

#### 2.7.6. Evaluation of Oxidative Stress Markers

Spectrophotometry was used to measure the levels of acetylcholinesterase (AchE). The assay combination had 0.05 millilitres of supernatant, 3 millilitres of a sodium phosphate buffer with a pH of 8, 0.10 millilitres of acetylthiocholine iodide, and 0.10 millilitres of DTNB (Ellman reagent). Spectrophotometrically, the shift in absorbance at 412 nm was instantly observable. The enzymatic activity in the supernatant was measured in µM/mg protein [82].

At pH 10.4, epinephrine was oxidized by SOD and spectrophotometrically measured for SOD activity. After adding 0.02 mL of epinephrine to the brain homogenate’s supernatant (0.2 mL), the mixture was brought to pH 10.4 using 0.8 mLof glycine buffer, 50 mM. Measurement of the absorbance at 480 nm was made after 5 min. nM/mg protein was used to measure SOD activity [83].

The 0.2 mLof tissue homogenate combined with 1.2 mL phosphate buffer was used to assess catalase activity (0.05 M, pH 7.0). Hydrogen peroxide (1.0 mL) was added to start the enzyme process (0.03 M). To monitor changes in absorbance, the enzyme blank was run with 1.0 mLof pure water instead of hydrogen peroxide for 3 min. The enzyme activity was measured in micromoles of hydrogen per minute per milligramme protein [84].

Brain homogenate was used for the quantitative analysis of malondialdehyde (MDA). The amount of MDA was measured at 532 nm with a spectrophotometer after its interaction with thiobarbituric acid, and the unit was reported as nM/mg protein [85].

The method described by Ellman et al., 1959was used to determine the brain’s reduced glutathione content. Sulfosalicylic acid (4%) was added to 1 mL of supernatant before being cold digested at 4 °C for an hour. For 15 min, the samples were spun at 1200 rpm in a centrifuge. The addition of phosphate buffer (0.1 M, pH 8) and 5,5′-dithiobis- (2-nitrobenzoic acid, DTNB) to 1 mLof the supernatant resulted in the formation of DTNB. At 412 nm, a spectrophotometer was used to measure the yellow colour that had formed. These data were represented in the supernatant in terms of mM/mg protein glutathione [86].

According to Green et al., 1982,Greiss reagent (0.1 percent N-(1-naphthyl) ethylenediamine dihydrochloride, 1 percent sulfanilamide, and 2.5 percent phosphoric acid) was used to determine the nitrite concentration in the supernatant. The supernatant and Greiss reagent were mixed and incubated for 10 min at room temperature in the dark, and the absorbance at 540 nm was measured. N-nitrosamine was measured using a sodium nitrite standard curve and expressed as µM/mg protein [84].

### 2.8. Protein Estimation

The protein content was quantified using the Coral protein estimation kit (Biuret method).

### 2.9. Gross Pathological Examination of Rat Brains

By decapitating and removing the animal’s brains, gross pathological analysis was performed over 36 days. The coronal sections were collected when the entire rat brain was observed [15,74]. On the glass, slides were inserted brain sections that had been coronally sectioned to a thickness of 2 millimetres (ranging from the anterior pole to the posterior poles of the cerebral cortex). In order to see all the brain regions, Fujifilm’s digital camera (Fujix digital camera) was employed. Demyelination regions (mm) in each brain segment were measured on day 43 of the operation using MOTICAM-BA310 image plus 2.0 software (Hong Kong, China). To compute the volume of the demyelination scale (mm), the demyelination region (mm) was converted to mm [1,87]. Image analysis on day 43 was used to evaluate each brain segment’s demyelination size (mm3) by focusing on the dark greyish area located around the striatum. Demyelination area (l × b × h) was used to estimate the extent of damage in 2-mmthick coronal sections of the brain [88,89].

### 2.10. Assessment of Histopathological Changes

The rats were anaesthetized with sodium phenobarbital (270 mg/mL, i.p.) and sacrificed by decapitation once the experimental regimen was completed. The mid-brain was carefully removed from the entire brain for histopathological examination. Before slicing into 0.5 µm slices, the isolated area was carefully cleaned. Following fixation in 4% paraformaldehyde in PBS, pH = 7.4, overnight at room temperature, the brain was treated with different grades of ethanol. The tissue was embedded in paraffin wax and then cut into small sections. After sectioning, different washing treatments with ethanol and xylene were performed. The tissue was then stained with hematoxylin and eosin dyes and observed under a florescence microscope [90].

#### 2.10.1. Assessment of Demyelination by Luxol Fast Blue

The tissue section was examined using luxol fast blue stain The dehydrated section was incubated in luxol fast blue overnight at 60 °C. The next day, the section was washed with 95% ethanol, followed by distilled water. The slide was immersed in lithium carbonate. The myelinated white matter traps the blue colour after lithium carbonate. The slide was rinsed with 70% ethanol until the grey matter turned white. After that, washing with distilled water was carried out, and the section was dehydrated with 95–100% ethanol. After that, the slide was cleaned with xylene three times for 10 min [91].

#### 2.10.2. Statistical Analysis

Data were analyzed using two-way ANOVA followed by posthoc Bonferroni test and one-way ANOVA repeated measures followed by posthoc test Tukey’s multi comparison test. *p* < 0.001 was considered statistically significant. Data were found to be normalized, and the sample size was calculated by checking the normality distribution by the Kolmogorov–Smirnov test. All statistical results were performed by GraphPad Prism version 5.03 for Windows (GraphPad Software, San Diego, CA, USA). Statistical results were expressed as the mean ± standard error of the mean (SEM).

## 3. Results

### 3.1. AKBA Mediated Neuroprotective Function on the Restoration of Body Weight in Experimental Model of Multiple Sclerosis

#### 3.1.1. Improvement in Body Weight after Long-Term Treatment with AKBA 

**Figure 2:**
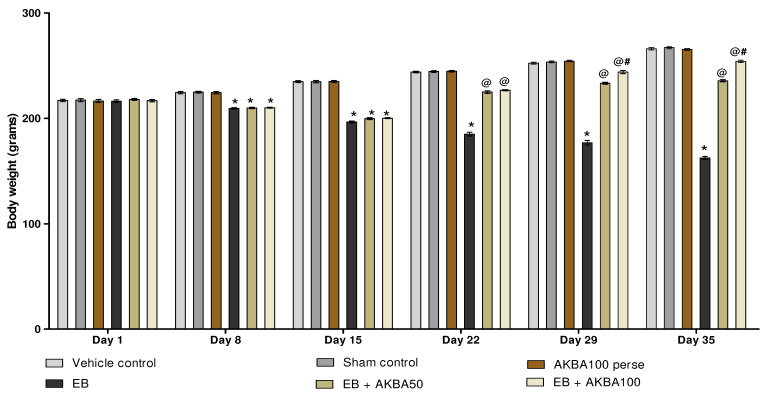
AKBA-mediated neuroprotective function on the restoration of body weight in an experimental model of multiple sclerosis. Statistical values are represented as mean ± SEM (*n* = 6 in each group). Statistical significant analysis was performed by posthoc Bonferroni test with two-way ANOVA. Here, * indicates versus vehicle control, sham control and AKBA100 perse group (*p* < 0.001); @ indicates versus EB group (*p* < 0.001); @# indicates versus EB + AKBA50 group (*p* < 0.001).

The body weight was measured on the 1, 8, 15, 22, 29, and 35 days. On the first day of the protocol schedule, all treatment groups had no significant change in body weight. After seven days of EB administration, the bodyweight of the rats gradually decreased in contrast to the vehicle-, sham-, and AKBA100 perse-treated rats. In comparison to the EB-injected MS rats, long-term oral administration with AKBA 50 mg/kg and 100 mg/kg resulted in a significant increase in body weight on the 22, 29, and 35days (two-way ANOVA: F(25,150) = 226.77, *p* < 0.001). AKBA 100 mg/kg-treated rats were more effective in restoring body weight than AKBA 50 mg/kg-treated rats on days 29 and 35 (Figure 2).

#### 3.1.2. AKBA-Mediated Restored Effect on the Relative Brain-Body Weight Ratio 

**Figure 3:**
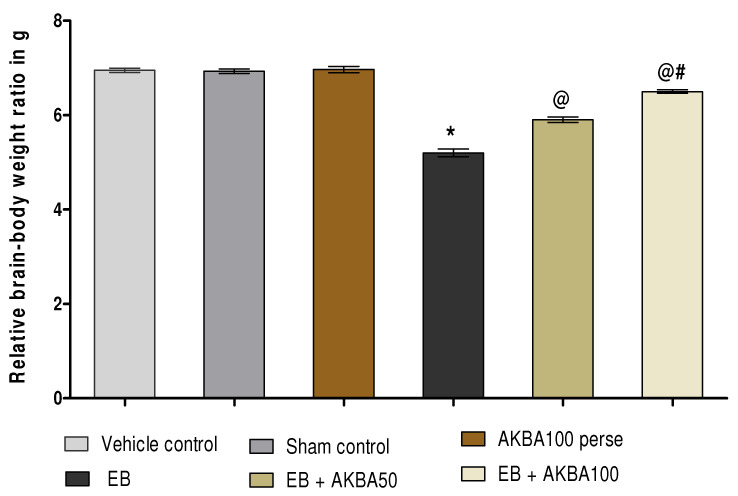
AKBA-mediated restored effect on relative brain-body weight ratio in an experimental model of multiple sclerosis. Statistical values are represented in mean ± SEM (*n* = 6 for each group). Statistical significant analysis was performed by posthoc Tukey’s test with one-way ANOVA. Here, * indicates versus vehicle control, sham control and AKBA100 perse group (*p* < 0.001); @ indicates versus EB group (*p* < 0.001); @# indicates versus EB + AKBA50 group (*p* < 0.001).

The ratio of the relative brain-body weight was calculated at the end of the experimental protocol. On day 35, non-significant variation was observed in the ratio of relative brain-body weight between all treatment groups. After regular gliotoxin EB injection, a significant reduction was observed in the ratio of relative brain-body weight as compared with vehicle, sham, and AKBA100 perse groups. Chronic administration with AKBA 50 mg/kg and 100 mg/kg orally significantly restored the ratio of relative brain-body weight on day 35 as compared to the EB-treated MS-rats (one-way ANOVA: F(5,25) = 1.027, *p* < 0.001). AKBA 100 mg/kg administered to rats restored the ratio of relative brain-body weight more efficiently than AKBA 50 mg/kg administered for 35 days (Figure 3).

### 3.2. AKBA-Mediated Neuroprotective Function on Behavioural Abnormalities in Experimental Model of Multiple Sclerosis

#### 3.2.1. Cognitive Improvement and Memory Consolidation after Long-Term Treatment of AKBA

**Figure 4:**
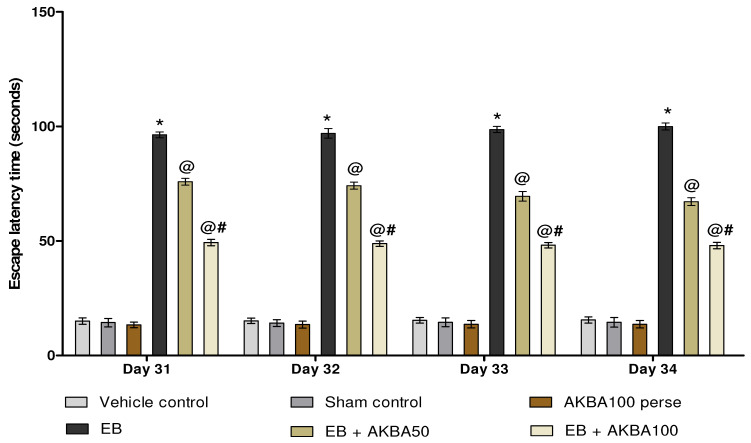
AKBA-mediated neuroprotective function on escape latency in an experimental model of multiple sclerosis. Statistical values are represented as mean ± SEM (*n* = 6 for each group). Statistical significant analysis was performed by posthoc Bonferroni’s test with two-way ANOVA. Here, * indicates versus vehicle control, sham control and AKBA100 perse group (*p* < 0.001); @ indicates versus EB group (*p* < 0.001); @# indicates versus EB + AKBA50 group (*p* < 0.001).

**Figure 5:**
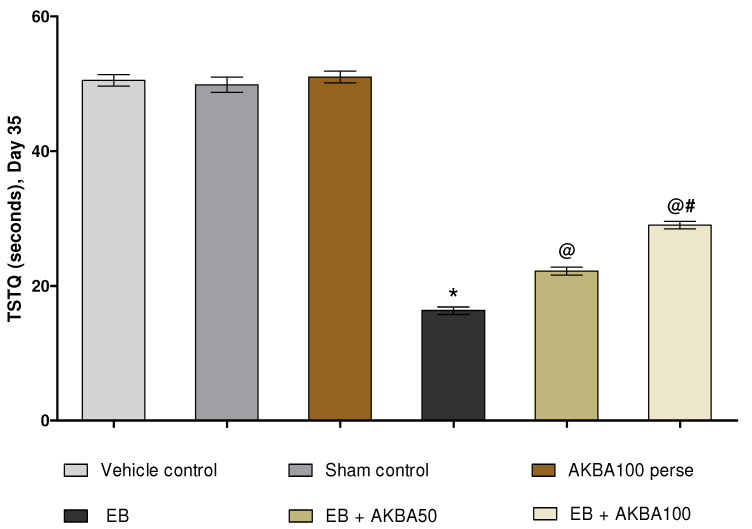
AKBA-mediated neuroprotective function on TSTQ in an experimental model of multiple sclerosis. Statistical values are represented as mean ± SEM (*n* = 6 for each group). Statistical significant analysis was performed by posthoc Tukey’s test with one-way ANOVA. Here, * indicates versus vehicle control, sham control and AKBA100 perse group (*p* < 0.001); @ indicates versus EB group (*p* < 0.001); @# indicates versus EB + AKBA50 group (*p* < 0.001).

Escape latency (ELT) was observed on days 31, 32, 33, and 34 of the experiment protocol schedule. EB-induced MS rats showed a substantial increase in ELT compared with vehicle-, sham-, and AKBA100 perse-treated rats. Prolonged oral treatment with AKBA 50 and 100 mg/kg substantially reduced ELT when compared to EB-injected MS rats (two-way ANOVA: F(15,90) = 8.80, *p* < 0.001). AKBA 100 mg/kg-treated rats were proven more effective in reducing ELT and improving memory and cognition than AKBA 50 mg/kg-treated rats (Figure 4). The time spent in the target quadrant (TSTQ) was recorded on day 35. Gliotoxin EB-injected MS rats showed a progressive reduction in TSTQ. At the same time, oral administration with AKBA 50 and 100 mg/kg significantly increased TSTQ compared to EB-treated MS rats (one-way ANOVA: F(5,25) = 0.567, *p* < 0.001). On the last day of the protocol, AKBA 100 mg/kg-treated rats were more capable of successfully increasing TSTQ and consolidating memory than the AKBA 50 mg/kg-treated rats (Figure 5).

#### 3.2.2. Improvement of Locomotor Activity after Long-Term Treatment with AKBA

**Figure 6:**
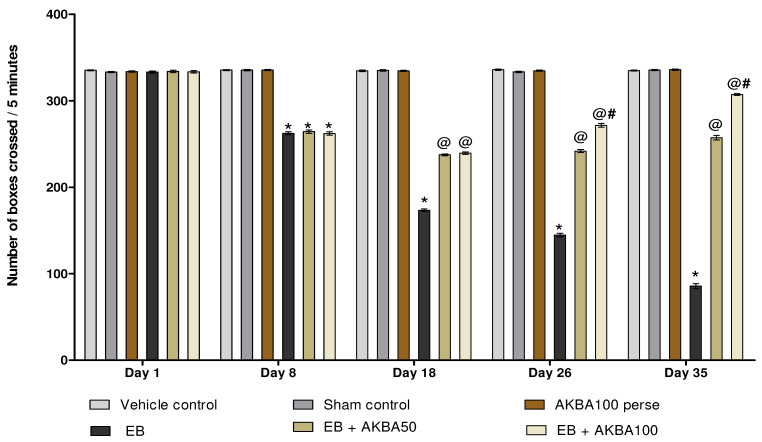
AKBA-mediated neuroprotective function on locomotor activity in an experimental model of multiple sclerosis. Statistical values are represented as mean ± SEM (*n* = 6 for each group). Statistical significant analysis was performed by posthoc Bonferroni’s test with two-way ANOVA. Here, * indicates versus vehicle control, sham control and AKBA100 perse group (*p* < 0.001); @ indicates versus EB group (*p* < 0.001); @# indicates versus EB + AKBA50 group (*p* < 0.001).

On days 1, 8, 18, 26, and 35, the effect of AKBA on locomotor activity was assessed. During the first day, all experimental groups had no significant change in locomotion. The rats treated with ED showed a significant decrease in locomotor activity on the eighth day, which continued throughout EB injection. Persistent treatment of AKBA 50 and 100 mg/kg orally improved locomotion on 18, 26, and 35 days as compared to the EB-induced MS-rats (two-way ANOVA: F(20,120) = 914.57, *p* < 0.001). On 26 and 35 days, AKBA 100 mg/kg was more potent and significantly improved locomotor activity more than the AKBA 50 mg/kg treatment group (Figure 6).

#### 3.2.3. Recovery of Gait Abnormalities after Long-Term Treatment of AKBA

**Figure 7:**
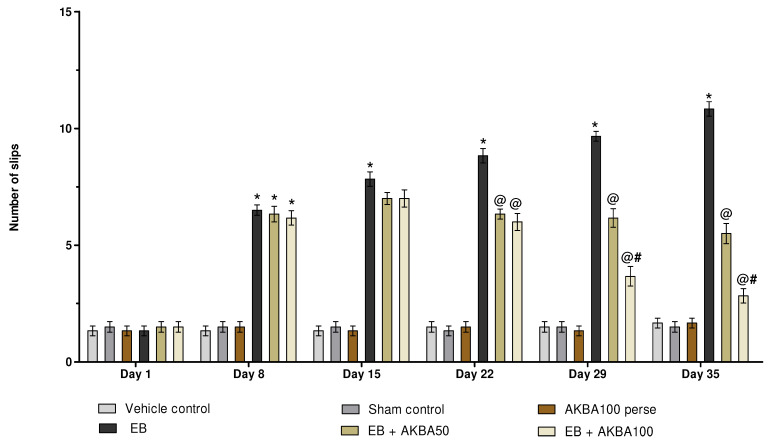
AKBA-mediated neuroprotective function on the number of slips in an experimental model of multiple sclerosis. Statistical values are represented as mean ± SEM (*n* = 6 for each group). Statistical significant analysis was performed by posthoc Bonferroni’s test with two-way ANOVA. Here, * indicates versus vehicle control, sham control and AKBA100 perse group (*p* < 0.001); @ indicates versus EB group (*p* < 0.001); @# indicates versus EB + AKBA50 group (*p* < 0.001).

The beam crossing task was performed on days 1, 8, 15, 22, 29, and 35. On day one, no significant changes were observed in the number of slips between all treated groups. The number of slips increased significantly after seven days of EB administration, which rose until the experimental schedule was completed. Long-term administration of AKBA 50 and 100 mg/kg showed a significant reduction in the number of slips on days 22, 29, and 35 compared with EB-induced MS rats (two-way ANOVA: F(25,150) = 35.02, *p* < 0.001). Especially compared to the AKBA 50 mg/kg treatment group, AKBA 100 mg/kg was found to be more successful in reducing the number of slips and improving gait abnormalities on days 29 and 35 (Figure 7).

#### 3.2.4. Improvement in Motor Coordination after Long-Term Treatment with AKBA

**Figure 8:**
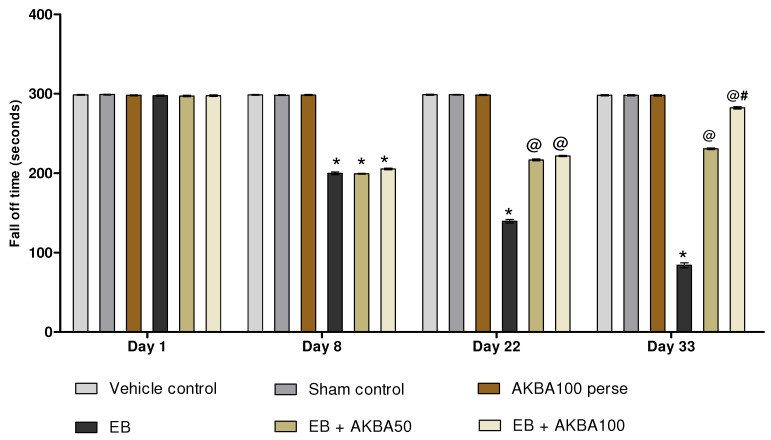
AKBA-mediated neuroprotective function on fall-off time in an experimental model of multiple sclerosis. Statistical values are represented as mean ± SEM (*n* = 6 for each group). Statistical significant analysis was performed by posthoc Bonferroni’s test with two-way ANOVA. Here, * indicates versus vehicle control, sham control and AKBA100 perse group (*p* < 0.001); @ indicates versus EB group (*p* < 0.001); @# indicates versus EB + AKBA50 group (*p* < 0.001).

Rotarod activity was performed on days 1, 8, 22and 33. On the first day, all treatment groups had no significant variation in fall-off time. The fall-off time (seconds) in EB-treated MS rats spontaneously increased on the eighth day. Chronic administration with AKBA 50 and 100 mg/kg significantly decreased the fall-off time on days 22and 33 as compared to EB-injected MS rats (two-way ANOVA: F(15,90) = 1142.13, *p* < 0.001). AKBA 100 mg/kg more efficiently decreased the fall-off time and restored motor coordination than AKBA 50 mg/kg for the 33 days (Figure 8).

### 3.3. AKBA-Mediated Neuroprotective Function on Neurochemical Alterations in Experimental Model of Multiple Sclerosis

#### 3.3.1. Increased Level of Nrf2 and HO-1 after Long-Term Treatment with AKBA

**Figure 9:**
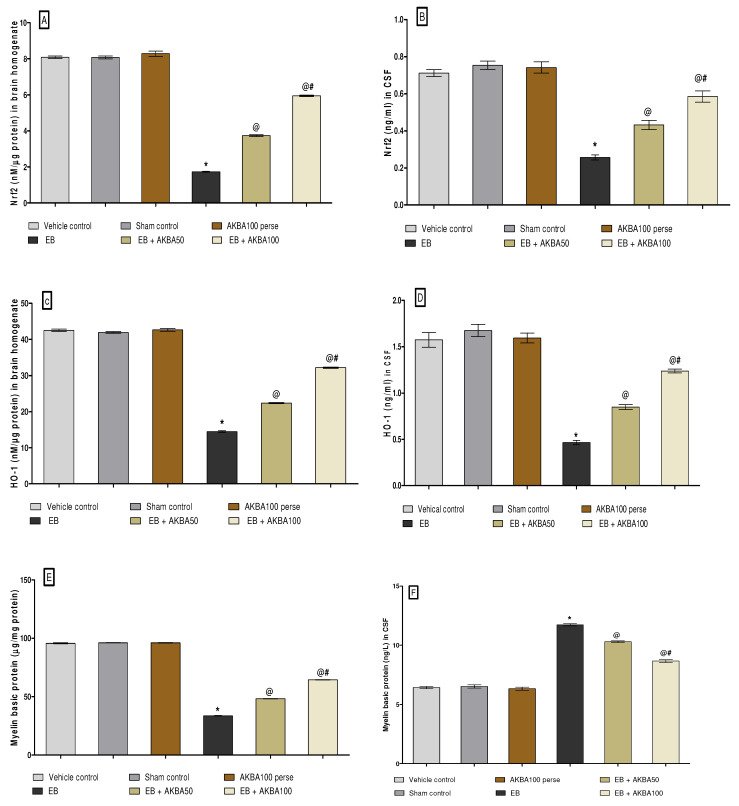
AKBA-mediated neuroprotective effect on Nrf2, HO-1 and myelin basic protein levels in an experimental model of multiple sclerosis. Statistical values are represented as mean ± SEM (*n* = 6 for each group). Statistical significant analysis was performed by posthoc Tukey’s test with one-way ANOVA. Here, * indicates versus vehicle control, sham control and AKBA100 perse group (*p* < 0.001); @ indicates versus EB group (*p* < 0.001); @# indicates versus EB + AKBA50 group (*p* < 0.001). (**A**): Nrf2 protein level in brain homogenate; (**B**): Nrf2 protein level in CSF; (**C**): HO-1 protein level in brain homogenate; (**D**): HO-1 protein level in CSF; (**E**): MBP protein level in brain homogenate ; (**F**): MBP protein level in CSF.

To determine whether AKBA is involved in the neuroprotective effect of the Nrf2/HO-1 transcription factor in EB-treated MS rats, the levels of Nrf2 and HO-1 were estimated in rat brain homogenate and CSF using an ELISA kit. EB-treated MS rats showed substantially decreased Nrf2 and HO-1 levels in the CSF and homogenate of the rat brain compared to the vehicle, sham, and AKBA 100 mg/kg perse treatment group. Prolonged oral treatment with AKBA 50 and 100 mg/kg substantially increased the level of Nrf2 in CSF sample (one-way ANOVA: F(5,25) = 0.365, *p* < 0.001) and homogenate of rat brains (one-way ANOVA: F(5,25) = 1.357, *p* < 0.001). Chronic oral administration with AKBA 50 and 100 mg/kg significantly increased the HO-1 level in rat brain homogenate (one-way ANOVA: F(5,25) = 2.376, *p* < 0.001) and CSF (one-way ANOVA: F(5,25) = 0.296, *p* < 0.001) as compared to the EB-induced MS rats. In contrast with the AKBA 50 mg/kg treatment group, the AKBA 100 mg/kg was more effective in restoring the Nrf2 and HO-1 protein levels than EB-induced MS rats (Figure 9A–D).

#### 3.3.2. Restoration of Myelin Basic Protein Level after Long-Term Treatment of AKBA

The levels of myelin basic protein (MBP) in CSF and the homogenate of rat brain were determined on the last day of the experiment. After chronic EB treatment, significantly decreased MBP levels in the homogenate of rat brain sand increased MBP levels in CSF was observed compared to the vehicle-, sham-, and AKBA100 perse-treatment group. Chronic oral treatment with AKBA 50 and 100 mg/kg led to a considerable increase in MBP levels in brain homogenates (one-way ANOVA: F(5,25) = 0.545, *p* < 0.001) Moreover, long-term oral treatment with AKBA 50 and 100 mg/kg substantially decreased the MBP levels in CSF (one-way ANOVA: F(5,25) = 0.923, *p* < 0.001) as compared to EB-treated MS rats. In contrast to the AKBA 50 mg/kg treatment group, AKBA 100 mg/kg effectively reduces MBP levels in CSF and restores MBP levels in brain homogenate (Figure 9E,F). 

#### 3.3.3. Decreased Caspase-3, Bax, and Increased Bcl-2 Levels after Long-Term Treatment with AKBA

The levels of apoptotic markers such as caspase-3, Bax and BCL-2 were determined in rat brain homogenate on the last of the experiment. Furthermore, substantial enhancements were observed in caspase-3, Bax, and Bcl-2 levels in the homogenate of rat brains after long-term administration of EB. Moreover, long-term EB-treated rats show a remarkable reduction in Bcl-2 protein levels in rat brain homogenates compared to vehicle-, sham-, and AKBA100-treated groups. Long-term oral administration with AKBA 50 mg/kg and 100 mg/kg considerably reduced caspase-3 levels in brain homogenate (one-way ANOVA: F(5,25) = 2.454, *p* < 0.001). Consequently, prolonged oral treatment of AKBA 50 mg/kg and 100 mg/kg substantially reduced the level of Bax in the homogenate of rat brains (one-way ANOVA: F(5,25) = 1.240, *p* < 0.001).

**Figure 10:**
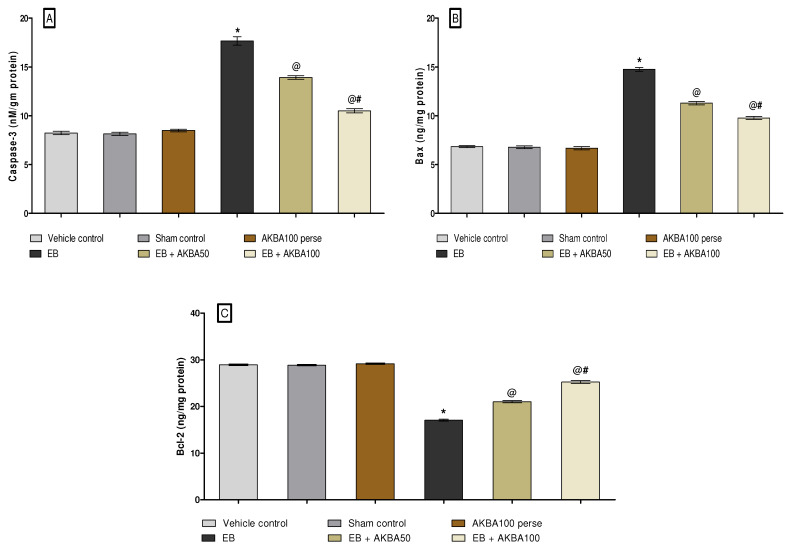
AKBA-mediated neuroprotective effect on caspase-3, Bax, and Bcl-2 levels in an experimental model of multiple sclerosis. Statistical values are represented as mean ± SEM (*n* = 6 for each group). Statistical significant analysis was performed by posthoc Tukey’s test with one-way ANOVA. Here, * indicates versus vehicle control, sham control and AKBA100 perse group (*p* < 0.001); @ indicates versus EB group (*p* < 0.001); @# indicates versus EB + AKBA50 group (*p* < 0.001). (**A**): Caspase-3 level in brain homogenate; (**B**): BAX level in brain homogenate; (**C**): Bcl-2 level in brain homogenate.

Moreover, chronic oral treatment of AKBA 50 mg/kg and 100 mg/kg showed significant increases in Bcl-2 levels in rat brain homogenate (one-way ANOVA: F(5,25) = 1.958, *p* < 0.001) as compared to EB-induced MS rats. AKBA 100 mg/kg treatment significantly enhances the levels of apoptotic markers more than AKBA 50 mg/kg treatment (Figure 10A–C).

#### 3.3.4. Restoration of Neurotransmitter Levels after Long-Term Treatment of AKBA 

**Figure 11:**
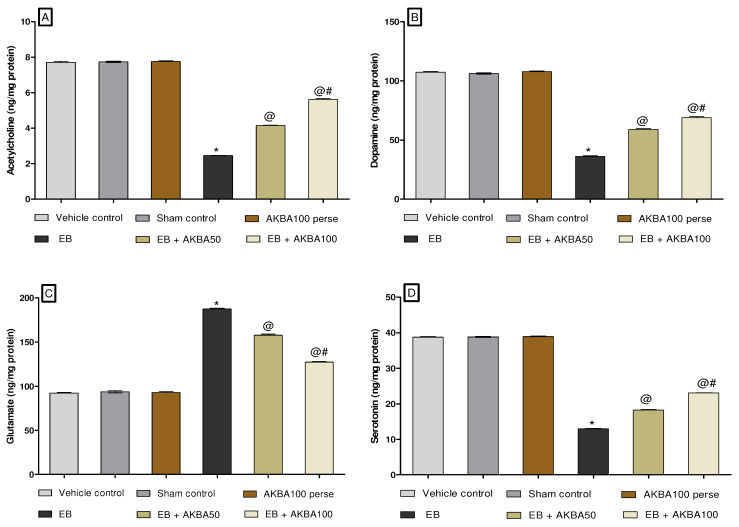
AKBA-mediated neuroprotective effect on amelioration of neurotransmitter levels in an experimental model of multiple sclerosis. Statistical values are represented as mean ± SEM (*n* = 6 for each group). Statistical significant analysis was performed by posthoc Tukey’s test with one-way ANOVA. Here, * indicates versus vehicle control, sham control and AKBA100 perse group (*p* < 0.001); @ indicates versus EB group (*p* < 0.001); @# indicates versus EB + AKBA50 group (*p* < 0.001). (**A**): Ach level in brain homogenate; (**B**): Dopamine level in brain homogenate; (**C**): Glutamate level in brain homogenate; (**D**): Serotonin level in brain homogenate.

At the end of the experiment, neurotransmitter levels such as acetylcholine, dopamine, glutamate, and serotonin were estimated in rat brain homogenate. Long-term EB administration leads to a substantial decrease in acetylcholine, dopamine, and serotonin. In contrast, an increased glutamate level was measured in rat brain homogenate compared with vehicle-, sham-, and AKBA100 perse-treated rats. Oral administration of AKBA 50 and 100 mg/kg considerably increased the levels of acetylcholine (one-way ANOVA: F(5,25) = 0.459, *p* < 0.001), dopamine (one-way ANOVA: F(5,25) = 1.877, *p* < 0.001), and serotonin (one-way ANOVA: F(5,25) = 1.156, *p* < 0.001). In comparison to EB-treated rats, there was a decrease in glutamate levels in rat brain homogenate (one-way ANOVA: F(5,25) = 0.485, *p* < 0.001). Furthermore, AKBA 100 mg/kg treatment more effectively improved the level of neurotransmitters compared to AKBA 50 mg/kg-treated rats (Figure 11A–D).

#### 3.3.5. Reduction in Inflammatory Cytokines Levels after Long-Term Treatment with AKBA

Neuro inflammatory cytokines such as TNF-α and IL-1β levels were examined in the homogenate of rat brain and blood plasma. Long-term treatment with EB in rats results in a considerable rise in inflammatory marketers such as TNF-α and IL-1β in rat brain homogenate and blood plasma compared to the vehicle-, sham-, and AKBA100 perse-treated group. Prolonged oral treatment with AKBA 50 and 100 mg/kg significantly decreased the level of the pro-inflammatory cytokine TNF-α in brain homogenate (one-way ANOVA: F(5,25) = 2.509, *p* < 0.001) and blood plasma (one-way ANOVA: F(5,25) = 0.200, *p* < 0.001).

**Figure 12:**
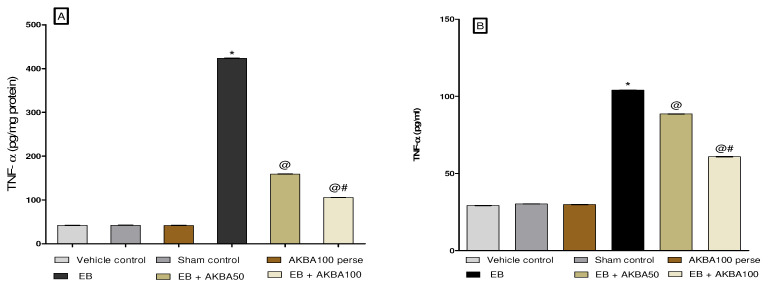

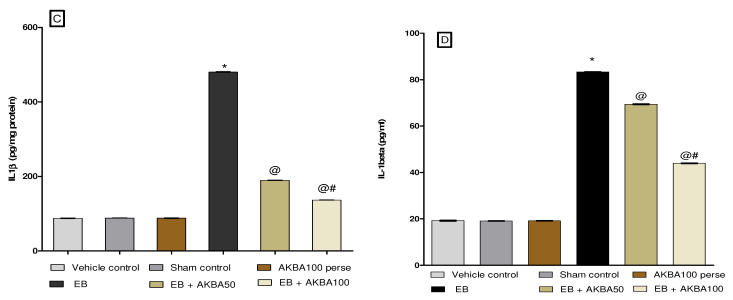
AKBA-mediated neuroprotective effect on the amelioration of inflammatory cytokines in an experimental model of multiple sclerosis. Statistical values are represented in mean ± SEM (*n* = 6 each group). Statistical significant analysis was performed by posthoc Tukey’s test with one-way ANOVA. Here, * indicates versus vehicle control, sham control and AKBA100 perse group (*p* < 0.001); @ indicates versus EB group (*p* < 0.001); @# indicates versus EB + AKBA50 group (*p* < 0.001). (**A**): TNF-α level in brain homogenate; (**B**): TNF-α level in blood plasma; (**C**): IL-1β in brain homogenate; (**D**): IL-1β in blood plasma.

However, long-term administration with AKBA 50 and 100 mg/kg orally gradually decreasedIL-1β levels in rat brain homogenate (one-way ANOVA: F(5,25) = 0.459, *p* < 0.001) and blood plasma samples (one-way ANOVA: F(5,25) = 2.868, *p* < 0.001) compared to EB-injected MS rats. As compared to the AKBA 50 mg/kg administered group, AKBA 100 mg/kg was more effective in reducing levels of inflammatory cytokines such as TNF-α and IL-1β (Figure 12A–D).

#### 3.3.6. Amelioration of Oxidative Stress Marker Levels after Long-Term Treatment with AKBA

**Figure 13:**
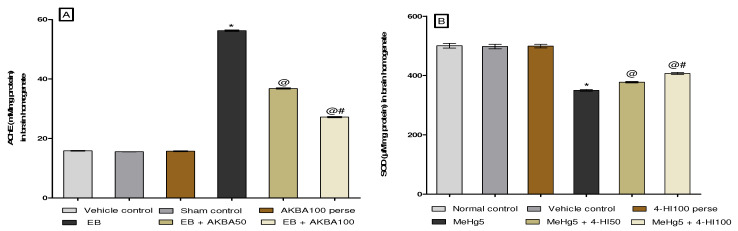

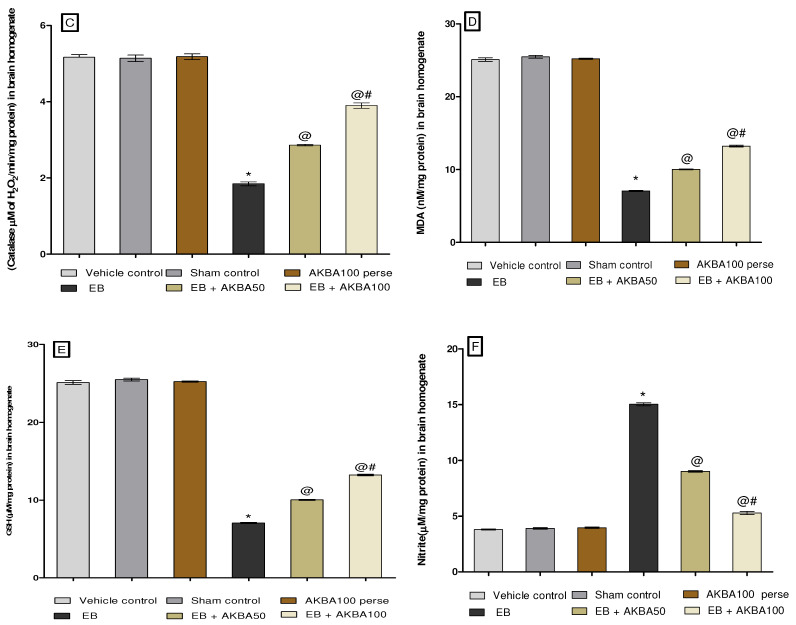
AKBA-mediated neuroprotective effect on oxidative stress markers in an experimental model of multiple sclerosis. Statistical values are represented in mean ± SEM (*n* = 6 for each group). Statistical significant analysis was performed by posthoc Tukey’s test with one-way ANOVA. Here, * indicates versus vehicle control, sham control and AKBA100 perse group (*p* < 0.001); @ indicates versus EB group (*p* < 0.001); @# indicates versus EB + AKBA50 group (*p* < 0.001). (**A**): Acetylcholinesterase level in brain homogenate; (**B**): SOD level in brain homogenate; (**C**): Catalase level in brain homogenate; (**D**): MDA level in brain homogenate; (**E**): Reduced glutathione level in brain homogenate ; (**F**): Nitrite level in brain homogenate.

Oxidative stress markers such as catalase, AchE, SOD, GSH, MDA, and nitrite were estimated in the rat brain homogenate on the experiment’s last day. The group receiving chronic EB treatment showed a massive increase in AchE, MDA, and nitrite levels. Moreover, substantially reduced GSH, SOD, and catalase levels were observed in contrast with the vehicle-, sham-, and AKBA100 perse-treated group. Administration with AKBA 50 and 100 mg/kg orally reduced AchE (one-way ANOVA: F(5,25) = 0.855, *p* < 0.001), MDA (one-way ANOVA: F(5,25) = 0.915, *p* < 0.001), and nitrite levels (one-way ANOVA: F(5,25) = 0.797, *p* < 0.001). Moreover, the level of reduced GSH (one-way ANOVA: F(5,25) = 0.540, *p* < 0.001), SOD (one-way ANOVA: F(5,25) = 1.475, *p* < 0.001), and catalase was significantly increased (one-way ANOVA: F(5,25) = 0.319, *p* < 0.001). AKBA 100 mg/kg more efficiently restores levels of antioxidant enzymes than the 50 mg/kg-administered group (Figure 13A–F).

### 3.4. AKBA-Mediated Neuroprotective Effect in the Restoration of Gross Pathological Alterations in Experimental Model of Multiple Sclerosis

#### 3.4.1. Restoration of Whole-Brain Alterations after Long-Term Treatment with AKBA

**Figure 14:**
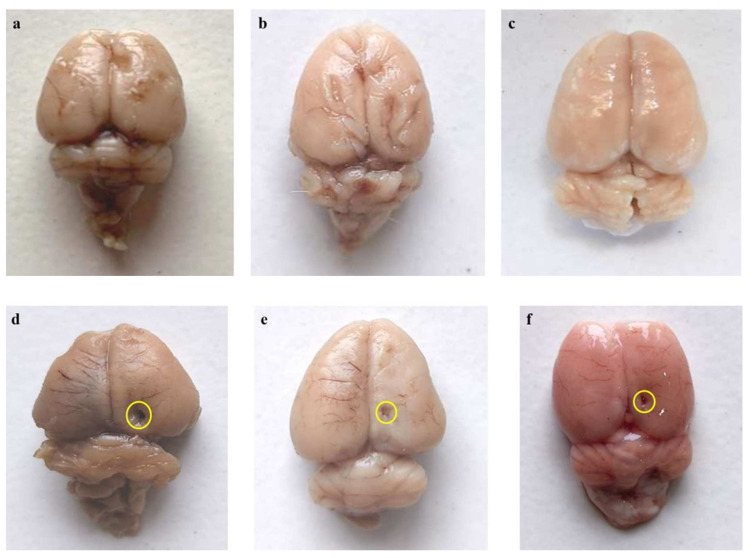
AKBA-mediated neuroprotective effect on the restoration of gross pathological alterations (whole rat brain) in an experimental model of multiple sclerosis. Vehicle control (**a**), sham control (**b**), AKBA100 perse (**c**), EB (**d**), EB + AKBA 50 (**e**), EB + AKBA 100 (**f**) (scale bar = 2 mm). Note: yellow circles show injury; *n* = 6 rats in each group.

The whole brains of rats treated with chronic EB displayed inflamed surfaces and depreciation in size. EB-treated rats were found to have breached meninges in the whole brain-injured region compared to the vehicle, sham, and AKBA100 perse treatment groups. In contrast with EB-administered MS rats, the brains of rats in the vehicle-, sham-, and AKBA100 perse-administered groups had appropriate sizes and shapes. Chronic administration of AKBA at 50 and 100 mg/kg orally restored morphological changes and helped the rat brains recover from further damage. Similarly, AKBA 100 mg/kg-treated rats showed more considerable improvement in the injured area of the brain and recovered brain damage compared to the AKBA 50 mg/kg treatment group (Figure 14).

#### 3.4.2. Reduction in Pathological Changes in Brain Sections after Long-Term Treatment with AKBA

**Figure 15:**
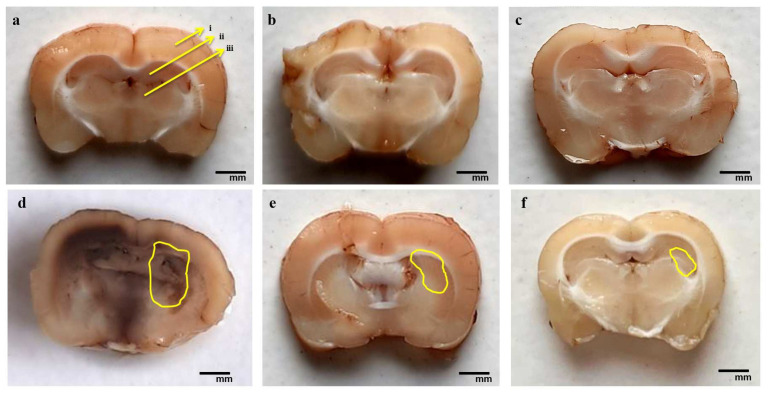
AKBA-mediated neuroprotective effect on the restoration of gross pathological alterations (brain section) in an experimental model of multiple sclerosis. (**a**) Vehicle control: (i) cerebral cortex, (ii) hippocampus, (iii) basal ganglia; (**b**) sham control; (**c**) AKBA100 perse; (**d**) EB; (**e**) EB + AKBA 50; (**f**) EB + AKBA 100 (scale bar = 5 mm). Note: yellow circles show injury; *n* = 6 rats in each group.

The EB-administered rat brain sections show a significant increase in white matter lesions in injured areas and a massive decrease in the size of basal ganglia, cortex, and hippocampal tissues. Compared to EB-induced rat brain sections, chronic oral administration of AKBA 50 and 100 mg/kg resulted in a considerable reduction in white matter damage, axonal loss, and restoration of the normal shape of basal ganglia, cortex, and hippocampal tissue. AKBA 100 mg/kg was more effective than AKBA 50 mg/kg in restoring the myelin sheath, enhancing recovery in white matter axons, and decreasing the necrosis volume of the basal ganglia, cortex, and hippocampal tissues. To validate this, we measured the demyelination volume and MBP levels in rat brain tissues (Figure 15).

#### 3.4.3. Reduction in Demyelination Volume after Long-Term Treatment with AKBA

**Figure 16:**
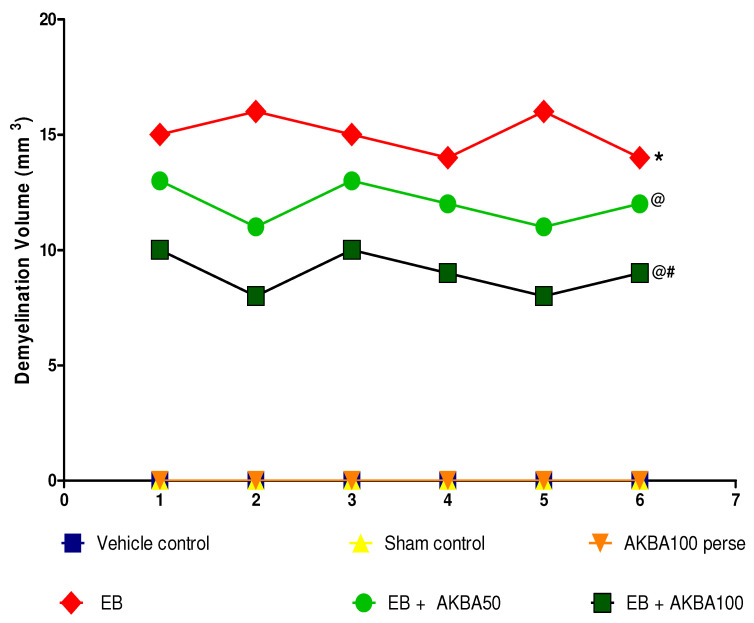
AKBA-mediated neuroprotective effect on demyelination volume in an experimental model of multiple sclerosis. Statistical analysis followed by one-way ANOVA (posthoc Tukey’s test). Values expressed as mean ± SEM (*n* = 6 rats per group). * *p* < 0.001 v/s vehicle control, sham control and AKBA100 perse; @ *p* < 0.001 v/s EB; @# *p* < 0.001 v/s EB + AKBA50. Note: Vehicle, Sham and Perse control groups were observed with no demyelination volume, therefore, volume in all three groups were expressed with value zero on X-axis.

The vehicle control and sham control groups showed no significant changes in demyelination volume and white matter integrity compared to the AKBA 100 mg/kg-treated group. On the other hand, long-term EB treatment significantly increased the white matter degradation of myelinated nerve fibres and the volume of demyelination compared to the vehicle-, sham-, and AKBA100 mg/kg perse-administered groups. Compared with EB-induced MS rats, prolonged oral treatment with AKBA 50 and 100 mg/kg significantly reduced white matter lesions and demyelination volume. AKBA 100 mg/kg effectively reversed white matter degeneration and considerably reduced demyelination in comparison to AKBA 50 mg/kg-treated rats, as further confirmed by MBP level assessment (one-way ANOVA: F(5,25) = 1.000, *p* < 0.001) (Figure 16).

### 3.5. AKBA-Mediated Neuroprotective Effect in Ethidium Bromide-Induced Histopathological Changes

**Figure 17:**
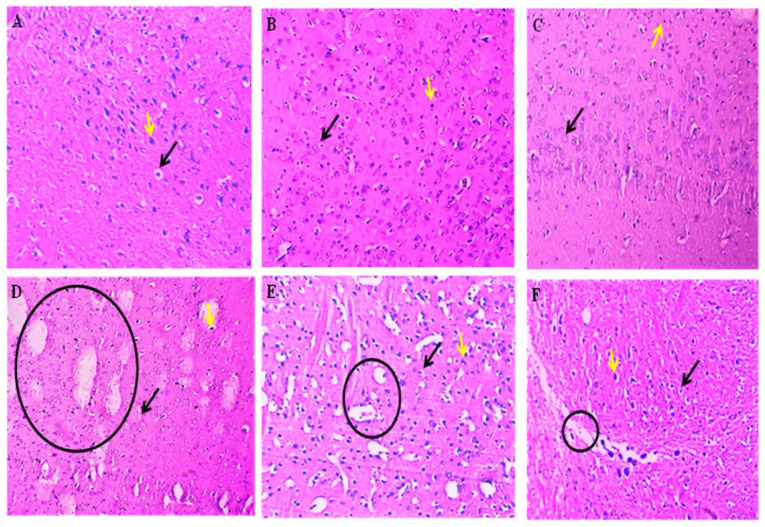
AKBA-mediated neuroprotective effect in ethidium bromide-induced histopathological changes of mid-brain by H&E staining. The vehicle (**A**), sham (**B**), and perse (**C**) groups showed typical neurons represented by the yellow arrow and normal oligodendrocyte numbers represented by the black arrow. The ethidium bromide (**D**) group showed a highly damaged area indicated by the black circle, aberrant neurons indicated by the yellow arrow and a decrease in the number of oligodendrocytes indicated by the black arrow. The EB + AKBA50 (**E**) and EB + AKBA100 (**F**) groups showed a decrease in the damaged area represented by the black circle, restoration of the oligodendrocytes indicated by the black arrow and neuronal cells represented by the yellow arrow in a dose-dependent manner.

The histological evaluation of the mid-brain by H&E staining revealed that the vehicle, sham and perse groups had normal neurons and normal oligodendrocyte numbers. Still, the ethidium bromide intoxication caused severe damage to the oligodendrocytes, thus reducing their number and adversely affecting the neurons, thereby causing an increase in the area of damage. However, the AKBA treatment dose-dependently showed a restorative effect on oligodendrocytes, resulting in both an increase in oligodendrocytes and improvement in the shape of the neurons, thus causing a decrease in the area of the damage. The AKBA100 treatment showed remarkable effects against ethidium bromide intoxication (Figure 17). 

### 3.6. AKBA-Mediated Neuroprotective Effect in Ethidium Bromide-Induced Demyelination

**Figure 18:**
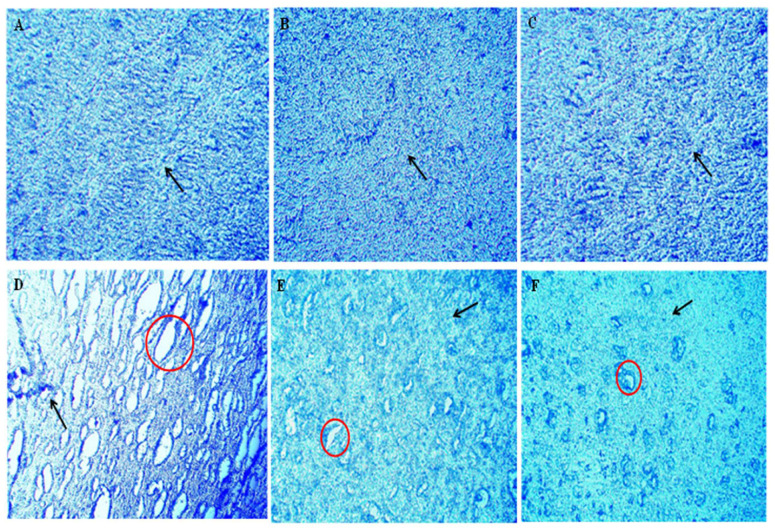
AKBA-mediated neuroprotective effect in ethidium bromide-induced demyelination using luxol fast stain. The mid brain was examined by luxol fast blue dye, which stains the myelin sheath. No damage was discovered to the myelin sheath in sections (**A**–**C**) of the luxol fast blue staining in the sham, vehicle, and AKBA perse groups, respectively. (Section (**D**)) displays the results of LFB staining seven days following ethidium bromide injection (disease group). The red circle highlights the demyelinated area, indicating that the oligodendrocytes have been destroyed. The black arrow in this image illustrates the damaged area surrounding the neurons. (Section (**E**)) LFB staining in the EB + AKBA50 group. The black arrow reflects a modest decrease in the demyelinated area, and the red circles show the destruction of axons caused by the destruction of the myelin sheath. In (Section (**F**)), for group EB + AKBA100, the black arrow reflects the damaged area that has been significantly reduced, and the demyelinated area can be seen, both indicative of the return of myelinated tissue.

Following a seven-day course of ethidium bromide injections, lesions and tissue loss indicated a demyelinated midbrain region. There were also significant interfascicular voids between the myelinated nerve fibres that were still intact. As seen in the images, AKBA was found to preserve axons and demonstrate various degrees of remyelination when administered at 50 and 100 mg/kg. Lesions in the demyelination area exhibited a modest decrease with AKBA 50 mg/kg. The demyelination region decreased markedly with AKBA 100 mg/kg, restoring the aberrant pattern of white matter bundles (Figure 18).

## 4. Discussion

The present research used the gliotoxin EB-treated experimental model of rats to examine the behavioral, molecular, and neuro chemical changes in MS rats. 

Our previous findings showed that EB injection via the ICP route might play an important role in the development and progression of MS [1,15]. Rats were exposed to the gliotoxin EB to induce demyelination [92]. EB-induced experimental models demonstrate extreme repeatability of demyelination in the specific region where demyelination was predefined [56,93]. Regular injections of EB for seven days resulted in complete demyelination by predominantly damaging glial cells, especially oligodendrocytes and astrocytes [1,94].

Evidence suggests that Nrf2/HO-1 signalling is upregulated by an AKBA, which has a beneficial influence on motor impairment, memory and cognitive deficits, neurochemical changes and aberrant behavior [36,55]. In the present study, we determined that AKBA exerts a protective effect via upregulating the Nrf2/HO-1 signalling pathway in EB-induced MS rats. Additionally, cellular and molecular markers, apoptotic indicators, neurotransmitters, pro-inflammatory cytokines, and oxidative stress were assessed in rat whole brain homogenate, blood plasma and cerebrospinal fluid.

Our behavioral findings indicate that administering 50 mg/kg and 100 mg/kg AKBA orally protects motor neuron dysfunction and neurological impairments in EB-induced experimental animals. Several behavioural and neurochemical parameters were measured to examine the potential role of AKBA on motor deficits caused by chronic administration of EB. Chronic EB administration showed a decreased body weight, but this was restored after giving AKBA in EB-induced rats. Similarly, AKBA treatment significantly restored the relative brain-body weight. The results of the Morris water maze (MWM) test were examined, and it was observed that AKBA therapy improved spatial memory in EB-treated rats. The escape latency time (ELT) in the MWM was markedly increased in EB-induced rats, although reduced TSTQ resulted in severe memory loss. AKBA treatment reduces ELT while significantly increasing TSTQ, indicating cognition improvement.

Additionally, ICP-EB led to considerable changes in several behavioural paradigms, such as the number of slips [94], locomotion activity [15], and balance and coordination [11], which were considered motor performance features. Oral administration of AKBA50 and 100 mg/kg restores motor balance and locomotion by lowering the number of slip counts, suggesting improved motor function. We also investigated AKBA’s potentialontheNrf2/HO-1 signalling cascade to explore a cellular signalling mechanism. In a recent study, a decrease in the levels of Nrf2/HO-1 was associated with the onset and progression of neurodegenerative disease [36,95]. Neuroinflammation is caused by Nrf2/HO-1 downregulation, which destroys neuroglia, primarily oligodendrocytes and astrocytes, resulting in myelin destruction [21]. In contrast, Nrf2/HO-1 upregulation was seen to have a protected effect on a patient suffering from MS [35,96]. 

As a result, we found that levels of Nrf2/HO-1 in CSF and brain homogenate of rats were decreased in EB-induced rats. AKBA treatment restored Nrf2 and HO-1 and their antioxidant, anti-inflammatory, and detoxifying protein levels in CSF and rat brain homogenate. Previous research showed that myelin basic protein levels were decreased, and myelin particles have also been observed in the perivascular space of MS subjects’ brains [97,98]. Pre-clinical studies have shown that MBP levels are reduced in EB-induced rat brains, which is a primary indication of MS [99]. A clinical study showed that myelin basic protein level was elevated in the cerebrospinal fluid of active RRMS patients [100,101]. MS’s start and development continues to be characterized by myelin sheath destruction. MBP is an important diagnostic marker in MS for identifying the degree of myelination [102,103].

In the present findings, the level of myelin basic protein decreased in brain homogenate and increased in cerebrospinal fluid upon exposure to EB. Additionally, in EB-treated rats, AKBA treatment restored myelin basic protein both in brain homogenate and cerebrospinal fluid. The results revealed that AKBA could either inhibit demyelination or accelerate remyelination. Additionally, Bax, Caspase-3 and Bcl-2 are essential in modulating apoptotic signaling [13]. Nrf2/HO-1 inactivation results in apoptosis [104]. Chronic EB treatment elevates the levels of pro-apoptotic markers while decreasing anti-apoptotic markers and brain homogenate. Long-term administration of 50 mg/kg and 100 mg/kg decreased apoptotic marker levels and restored anti-apoptotic marker levels in EB-treated rats in different biological samples.

Regarding neurotransmitters, glutamate activation in OPCs was associated with oligodendrocyte death. It has been proven that it promotes white matter degradation, whereas dopamine, acetylcholine, and serotonin alter neural excitability, producing a remarkable alteration in mood and attention [105,106]. According to the analysis, in the EB-treated group, the levels of serotonin, acetylcholine, and dopamine were decreased, while glutamate levels were increased. AKBA could restore levels of dopamine, serotonin, and acetylcholine and reduce levels of glutamate in the brain homogenates of rats. The levels of inflammatory cytokines such as TNF-α and IL-1 in the brain homogenate sand blood plasma were increased by long-term administration of EB. However, prolonged treatment with AKBA 50 and 100 mg/kg orally substantially reduced inflammatory cytokine levels for TNF-α and IL-1β in rat brain homogenate and blood plasma.

The progression of MS is initiated when Nrf2/HO-1 is downregulated, resulting in increased oxidative stress [22,107]. The previous finding revealed that upregulation of the Nrf2/HO-1 antioxidant signaling cascade showed a neuroprotective effect by reducing oxidative stress [108]. Our findings demonstrated that EB treatment markedly elevated MDA, AchE, and nitrite levels while significantly lowering the levels of antioxidant enzymes such as GSH, catalase, and SOD. Chronic AKBA dosing decreased AchE, MDA, and nitrite levels in brain homogenate while elevating the levels of antioxidant enzymes like catalase, SOD, and GSH.

Earlier clinical research indicated that postmortem MS brains have degeneration of cortical grey and white matter lesions. In addition, patients with MS often have cerebral demyelination and the death of neurons [109,110]. Axonal degeneration and brain atrophy are noticed in MS’s primary phase. Our previous findings indicate that EB injection caused a reduction in brain size and a gradual increase in demyelination volume in experimental animals [1]. In the present research, administration of EB resulted in a decreased brain size, myelin sheath destruction, increased demyelination, and white matter deterioration in the basal ganglia, hippocampus, and cortex tissues. Chronic AKBA treatment in EB-induced rats restored brain size, recovered the myelin sheath, and decreased white matter degeneration. These findings indicate that AKBA can inhibit demyelination while also promoting remyelination. AKBA’s protective effect was also evaluated by histological (H&E) and LFB staining techniques. Histological examination of the mid-brain revealed that ethidium bromide intoxication results in a severe decrease in the number of oligodendrocytes and adversely affects the shape of neurons. Our histological examination of the mid-brain showed that ethidium bromide caused a decrease in oligodendrocytes and neurons, thus resulting in an increase in the damaged area, which complies with the previous reports. However, AKBA treatment protected oligodendrocytes and neurons against ethidium bromide-induced histological alterations and ultimately decreased the area of damage, indicating the protective effect of AKBA against ethidium bromide. LFB staining is used to detect myelinated and demyelinated areas. LFB slide examination showed that seven-day ethidium bromide injections caused lesions and tissue loss, indicating a demyelinated midbrain region. There were also significant interfascicular voids between the myelinated nerve fibres that were still intact. AKBA was found to preserve axons and demonstrate various degrees of remyelination when administered at 50 mg/kg and 100mg/kg. Lesions in the demyelination area exhibited a modest decrease in AKBA 50 mg/kg. The demyelination region decreased markedly with AKBA 100 mg/kg, restoring the aberrant pattern of white matter bundles.

Furthermore, the findings of this study are only correlations in which AKBA was mainly explored to improve behavioral and neurochemical deficits in EB-induced MS rats via the Nrf2/HO-1 signalling pathway. Our findings imply that Nrf2/HO-1 and MBP levels in brain homogenate, blood plasma, and CSF can be used as an earlier stage diagnostic marker to detect major degenerative characteristics of the MS brain.

Moreover, knock-in/knock-out experiments of the Nrf2 and HO-1 genes are required to validate this mechanistic approach. Furthermore, additional studies for cellular markers, such as Western blot and immunohistochemistry, are expected to validate these assumptions. Concurrent studies, such as Western blot and immunohistochemistry, are also necessary to offer molecular evidence for this idea. Despite these limitations, the protective effect of AKBA in correcting or upregulating the aberrant functioning of the Nrf2/HO-1 signalling cascade in the CNS seems promising.

## 5. Conclusions

Our research findings suggest that AKBA is involved intheNrf2/HO-1 signalling pathway and influences behavioural and neurochemical alterations caused by EB injection. Until now, no pre-clinical research on the protective role of AKBA through upregulation of the Nrf2/HO-1 pathway in EB-induced experimental MS rats has been carried out. AKBA may be a potential treatment choice for improving different behavioral, neurochemical, and morphological changes involved in MS pathogenesis. Levels of apoptotic protein (caspase-3, Bax, Bcl-2) and inflammatory cytokines (TNF-α and IL-1β) were also examined in the plasma blood of rats. The current results indicate that AKBA suppressed demyelination, initiated the remyelination process, and increased the levels of MBP in the brains of EB-induced experimental rats. Furthermore, AKBA was able to reverse the gross pathological changes in both the brain section and whole brain, illustrating that it has a protective effect against EB-treated motor abnormalities. Our findings suggest that Nrf2/HO-1 may be used as a diagnostic marker in the initial stage of MS and neurological diseases. Moreover, additional Western blots and immunohistological analyses are required to elucidate the mechanisms regulating such interrelationships. Activation of Nrf2 and HO-1 may be useful as future treatment strategies for several diseases.

## Data Availability

All data generated or analyzed during this study are included in this article. There are no separate or additional files.

## Abbreviations

Ach	Acetylcholine
AchE	Acetylcholinesterase
AD	Alzheimer’s disease
AKBA	Acetyl-11-keto-β-boswellic acid
ALS	Amyotrophic lateral sclerosis
ARE	Antioxidant response element
BCT	Beam crossing task
CNS	Central nervous system
CSF	Cerebrospinal fluid
EB	Ethidium bromide
ELISA	Enzyme-linked immunosorbent assay
ELT	Escape latency time
GABA	γ-aminobutyric acid
GSH	Glutathione
HD	Huntington’s disease
HO-1	Heme oxygenase-1
ICH	Intracerebral hemorrhage
ICP	Intracerebropeduncle
IL-1 β	Interleukin-1 β
Keap1	Kelch-like ECH-associated protein-1
MBP	Myelin basic protein
MDA	Malondialdehyde
MS	Multiple sclerosis
MWM	Morris water maze
NO	Nitric oxide
Nrf2	Nuclear factor erythroid-2-related factor-2
PD	Parkinson’s disease
ROS	Reactive oxygen species
RT-PCR	Reverse transcription polymerase chain reaction
SOD	Superoxide dismutase
TNF-α	Tumor necrosis factor-α
TSTQ	Time spent in the target quadrant
